# The Medical Record Visiting List

**Published:** 1881-11-20

**Authors:** 


THE Medical Record Visiting List or Physician's Diary, for 1881, is kindly sent us by Wm. Wood & Co., 17 Great Jones St., New York. Exceedingly convenient and useful to the practitioner. Furnished in two series, the one for 30 patients at $1.25, the other for 60 patients at $1.50. It contains tables of weights and measures, both the old style and the metric system—list of drugs and doses, disinfectants, poisons and antidotes, and other useful and instructive matter.

